# MicroRNA in Lung Cancer Metastasis

**DOI:** 10.3390/cancers11020265

**Published:** 2019-02-23

**Authors:** Shang-Gin Wu, Tzu-Hua Chang, Yi-Nan Liu, Jin-Yuan Shih

**Affiliations:** 1 Department of Internal Medicine, National Taiwan University Hospital, National Taiwan University, Taipei 10002, Taiwan; b8501091@gmail.com (S.-G.W.); thchang15@gmail.com (T.-H.C.); benson1032@gmail.com (Y.-N.L.); 2 Department of Internal Medicine, National Taiwan University Cancer Center, National Taiwan University, Taipei 10672, Taiwan; 3 Graduate Institute of Clinical Medicine, College of Medicine, National Taiwan University, Taipei 10002, Taiwan

**Keywords:** microRNA, metastasis, lung cancer, epithelial-to-mesenchymal transition

## Abstract

Tumor metastasis is a hallmark of cancer, with distant metastasis frequently developing in lung cancer, even at initial diagnosis, resulting in poor prognosis and high mortality. However, available biomarkers cannot reliably predict cancer spreading sites. The metastatic cascade involves highly complicated processes including invasion, migration, angiogenesis, and epithelial-to-mesenchymal transition that are tightly controlled by various genetic expression modalities along with interaction between cancer cells and the extracellular matrix. In particular, microRNAs (miRNAs), a group of small non-coding RNAs, can influence the transcriptional and post-transcriptional processes, with dysregulation of miRNA expression contributing to the regulation of cancer metastasis. Nevertheless, although miRNA-targeted therapy is widely studied in vitro and in vivo, this strategy currently affords limited feasibility and a few miRNA-targeted therapies for lung cancer have entered into clinical trials to date. Advances in understanding the molecular mechanism of metastasis will thus provide additional potential targets for lung cancer treatment. This review discusses the current research related to the role of miRNAs in lung cancer invasion and metastasis, with a particular focus on the different metastatic lesions and potential miRNA-targeted treatments for lung cancer with the expectation that further exploration of miRNA-targeted therapy may establish a new spectrum of lung cancer treatments.

## 1. Introduction

Lung cancer constitutes the leading cause of cancer death worldwide [1], with most patients presenting advanced disease stage at initial diagnosis. Although early screening by computed tomography (CT) reduces the associated mortality [2], tumor invasion and migration-mediated disease progression represents the leading cause of cancer-related death despite standard treatment.

Numerous studies regarding tumor invasion and migration depict the interaction between tumor cells and adjacent tissues or microenvironments, reporting different mechanisms and various signal pathways related to tumor spreading. Specifically, the critical role of the epithelial-to-mesenchymal transition (EMT) in cancer invasion, migration, and metastasis provides a clue to prevent cancer spread and identify possible therapeutic targets [3].

MicroRNAs (miRNAs), a group of small non-protein-coding RNAs (20–25 nucleotides), suppress gene expression primarily through direct interaction with the 3′-untranslated region (3′UTR) of corresponding target messenger RNAs (mRNAs) [4]. Target mRNA fate depends on the seed match architecture between the miRNA binding and mRNA seeding sequences. mRNA degradation is induced upon perfect miRNA complementary with the seeding sequence, whereas imperfect or partial complementarities effect protein translational suppression [4]. The imperfect matching and relatively short paired seeding sequences allow miRNAs to regulate various target mRNAs. Identifying different miRNA expression patterns thus supports tissue-specific miRNA classification, and disease status and outcome prediction [5,6]. Aberrantly expressed miRNAs in different malignancies function as tumor suppressors or oncomirs [7,8], regulating cancer biology by controlling target mRNA expression to facilitate tumor growth, invasion, angiogenesis, and immune evasion [9,10]. Here, we review current findings regarding the role of miRNAs in lung cancer invasion and metastasis, focusing on the different metastatic lesions and potential miRNA-targeted treatments.

## 2. EMT: The Key Mechanism of Lung Cancer Metastasis

**Figure 1:**
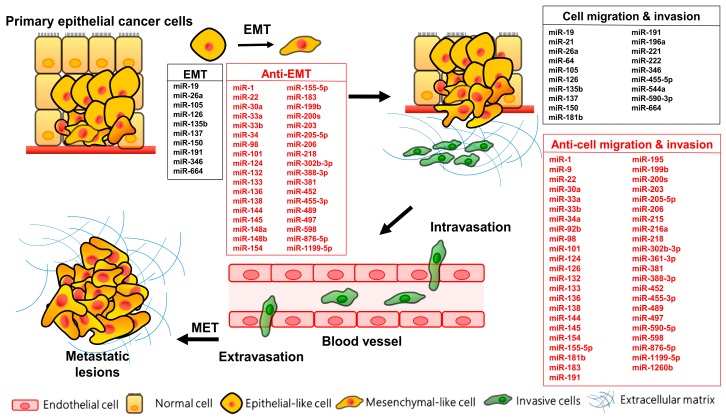
MicroRNAs involved in distinct steps of metastasis including EMT and migration/invasion in lung cancer. EMT: epithelial-to-mesenchymal transition; MET: mesenchymal-to-epithelial transition.

Distant spreading of the primary tumor represents the major cause of cancer-related deaths in non-small cell lung cancer (NSCLC), especially metastasis to the brain [11,12]. Metastasis is a complex process by which cancer cells spread from a localized lesion to systemic disease. The “metastatic cascade” includes tumor cells surmounting physical boundaries, basement membrane and surrounding tissue invasion, entry into the blood/lymphatic stream (migration and intravasation), extravasation at the secondary sites, and proliferation [13,14]. EMT encompasses the various molecular factors, phenotype changes, and genetic alterations in the multistep dissemination process [15,16]. Conversely, cell spread and proliferation as metastatic lesions requires a mesenchymal-to-epithelial transition [17] (Figure 1).

In epithelial-to-mesenchymal transition (EMT), uncontrolled epithelial cells first reduce dependence on their normal tissue microenvironment and proliferate [18]. Polarized epithelial cells lose epithelial cell junctional proteins, such as E-cadherin, claudins, and zona-occludens 1 (ZO-1), gaining mesenchymal markers including N-cadherin, vimentin, and fibronectin, with cytoskeletal reorganization. EMT, which engages different molecular process, transcription factor (TF) activation, and alternative miRNA expression [16], occurs in various physiological or pathological processes and is categorized according to biological and functional consequences: fertilized oocyte implantation and embryonic gastrulation (Type 1); inflammation and fibrosis (Type 2); and cancer cell invasion and metastasis (Type 3) [19]. In cancers, epithelial malignant cells acquire mesenchymal characteristics to promote migratory capacity, invasiveness, resistance against apoptosis, and extracellular matrix (ECM) component production [16,20].

Numerous genetic and epigenetic alternations are associated with type 3 EMT, which is driven by intrinsic oncogenic activation (e.g., Kirsten rat sarcoma viral oncogene homolog (KRAS), human epidermal growth factor receptor 2 (Her2), hepatocyte growth factor receptor (MET), and epidermal growth factor receptor (EGFR)) [21,22,23,24,25,26] or external microenvironmental stimuli (myofibroblasts, cancer-associated fibroblasts, infiltrating immune cells, ECM, growth factors (transforming growth factor-β (TGF-β), EGF, hepatocyte growth factor (HGF), and platelet-derived growth factor (PDGF)), and cytokines (tumor necrosis-α (TNF-α) and interleukin-6 (IL-6)) [3,16]. Cell surface proteins, such as αV integrins, also induce EMT by activating TGF-β1 [27]. Such microenvironmental stromal cell-produced factors activate diverse signaling pathways including TGF-β/bone morphogenic protein (BMP), wingless-type murine mammary tumor virus (MMTV) integration site family (WNT)/β-catenin, Sonic hedgehog (SHH), Notch, and phosphoinositide-3-kinase/protein kinase B (PI3K/AKT) [16], along with EMT-inducing TFs, such as Snail gene family (Snail and Slug), zinc finger E-box binding homeobox 1 (ZEB1), ZEB2 (Smad interacting protein 1 (SIP1)/ZFXH1b), lymphoid enhancer binding factor (LEF-1), Twist, and forkhead Box C2 (FOXC2) [3,16,28,29,30]. EMT initiation leads associated intracellular signaling network activation, involving signal-transducing proteins, PI3K, AKT, Ras, mitogen-activated protein kinase (MAPK), extracellular-signal-regulated kinase (ERK), Smads, Ras Homolog Family Member B (RhoB), c-Fos, β-catenin, and lymphoid enhancer binding factor (LEF).

## 3. MicroRNAs Regulate EMT in Lung Cancer

Accumulating evidence suggests that miRNAs comprise key regulators to control EMT signaling pathways and TFs. Because some miRNAs directly target EMT-TF, miRNAs and EMT-TF form tightly interconnected negative feedback loops that regulate the expression of TF, epithelial cell plasticity, and cell invasion/migration. Therefore, alterations in miRNAs expression have impacts on EMT program and metastasis cascade in cancer [31]. Although it is still poorly known about the regulation of miRNAs expression linked to EMT, more and more signal pathways and mechanisms are explored and clarified. Various miRNAs also regulate EMT in NSCLC (Figure 1 and Table 1).

### 3.1. SNAI Family TF-Related miRNAs

The Snail gene family encodes three TFs: SNAI1 (also designated as SNAIL), SNAI2 (SLUG), and SNAI3 (SMUC). Their activation down-regulates epithelial gene expression including E-cadherin, and up-regulates that of mesenchymal genes; e.g., N-cadherin, β-catenin, and fibronectin [32]. Various miRNAs regulate EMT by directly targeting the Snail family.

The p53/miR-34 axis regulates Snail expression in different cancer cell lines including lung cancer. p53 knockdown induces SNAIL protein expression by down-regulating miR-34 expression [33]. MiR-126 regulates lung cancer cell invasion and migration in vitro and in vivo, suppressing EMT by directly targeting PI3K/AKT/Snail signaling [34]. MiR-346, which is up-regulated in NSCLC compared with adjacent normal lung tissues, acts as an oncogenic miRNA to promote cell proliferation, metastasis, and decrease cell apoptosis by regulating the xeroderma pigmentosum, complementation group C (XPC)/ERK/SNAIL/E-cadherin pathway [35]. MiR-22 and miR-30a, which are down-regulated in NSCLC, directly target Snail to regulate EMT [36,37,38]. Their overexpression in lung cancer cell lines suppresses invasion and migration and attenuates EMT, including increased E-cadherin expression and decreased N-cadherin level. Finally, miR-381 targets both Snail and Twist [39].

MiR-137 promotes lung cancer invasion and metastasis by suppressing transcription factor AP-2 gamma (TFAP2C). Patients harboring lung adenocarcinoma with low-level Slug and miR-137 albeit high-level TFAP2C expression exhibit significantly longer overall survival (OS) [53]. MiR-124 regulates NSCLC cell invasion by translationally suppressing Slug and modulates resistance to gefitinib, an EGFR tyrosine kinase inhibitor (TKI) [46,47]. As a tumor suppressor; miR-218 expression level inversely correlates with advanced stage and lymph node metastasis. MiR-218 overexpression in lung cancer cell lines induces higher E-cadherin and lower vimentin expression levels. It inhibits EMT, tumor cell migration, and invasion by directly targeting Slug [59].

MiR-1 also directly targets Slug to regulate EMT. MiR-1 overexpression in A549 lung cancer cells causes significant morphological change from mesenchymal to epithelial phenotype with increased E-cadherin, attenuating invasion and migration [40]. Vascular endothelial growth factor-A (VEGF-A) induces sex determining region Y-box 2 (Sox2) to drive stem cell expansion by down-regulating miR-452, which directly targets the SLUG 3′UTR to suppress metastasis, causing Slug up-regulation [64].

### 3.2. ZEB1/ZEB2 TF-Related miRNAs

ZEB1/ZEB2, well-characterized EMT TFs, mediate cell plasticity, metastasis, and treatment resistance in different cancers [107,108,109]. ZEB1/ZEB2 repress E-cadherin transcription by binding to E-box sites in the ZEB1/ZEB2 promoters via their zinc finger motifs. ZEB1 overexpression in normal immortal human bronchial epithelial cells directly suppresses epithelial splicing regulatory protein 1 expression, thereby up-regulating mesenchymal CD44 splice variant expression to elicit a more invasive phenotype [110].

The widely studied miR-200 family is crucial in cancer initiation and metastasis [111]. Their promoter regions are bound by the TFs ZEB1, ZEB2, p53, specificity protein-1 (Sp1), and Wnt inhibitory factor 1 [112]. Upon promoter binding, ZEB1 and ZEB2 inhibit miR-200 family transcription, whereas p53 and Sp1 activate miR-200b/200a/429 and miR-200c/141 cluster transcription, respectively [81,112,113,114]. The highly invasive lung cancer cells H1299, A549, and SPC-A-1sci are characterized by lower miR-200c expression levels. MiR-200c inhibits invasion and metastasis by directly targeting ubiquitin specific peptidase 25 (USP25) [115]. Up-regulated miR-200c in A549 cells alters cell morphology and causes ZEB1 loss with increase of its regulatory target, E-cadherin [116]. MiR-200c dysregulation is associated with tumor progression, EMT, and drug resistance [117,118]. Notably, the miR-200 family also directly suppresses ZEB1 in a negative feedback loop; this reciprocal repression stabilizes EMT and promotes invasion [81]. MiR-200/ZEB1 axis-related EMT is also associated with antitumor immunity suppression, with the EMT regulatory axis controlling PD-L1 expression on tumor cells. PD-L1 inhibitors, a kind of immune checkpoint inhibitor, may thus comprise treatment options for the subgroups of patients exhibiting malignant progression driven by EMT activators [119]. 

MiR-205 (miR-205-5p) also suppresses EMT by targeting the ZEB1 and ZEB2 3′UTR [74]. Patients with NSCLC exhibiting low miR-205 expression had shorter relapse-free survival than those with high expression [120]. MiR-205 depletion releases the ZEB1 and SRC suppression (as both are miR-205 targets) and induces EGFR tyrosine kinase inhibitor resistance in EGFR mutant lung cancer [121].

MiR-455, a tumor suppressor, is significantly down-regulated in NSCLC tumor tissue samples and cell lines. MiR-455 (miR-455-3p) up-regulation inhibits cell proliferation, invasion, and migration by directly targeting ZEB1 [91]. Restoring ZEB1 rescues miR-455-induced suppression of tumor progression. MiR-33b inhibits cell growth, invasion, and EMT by suppressing Wnt/β-catenin/ZEB1 signaling [41]. MiR-1199-5p and ZEB1 form a reciprocal repressive feedback loop to potentially coordinate EMT and tumor metastasis [95]. MiR-101, miR-155-5p, and miR-199b, as tumor suppressors, inhibit EMT by targeting ZEB1 [54,67,77]. In turn, ZEB1 inactivates miRNAs by up-regulating competing RNAs (ceRNAs) or silencing the expression of TFs that drive miRNA expression [48,73]. The competing RNA-integrin α1 (ITGA1) competes with adenylyl cyclase 9 (ADCY9) for miR-181b. ZEB1 up-regulates ITGA1 to recruit miR-181b, thus relieving ADCY9 to drive metastasis in lung cancer cells [73]. ZEB1 also directly controls miR-203 and miR-200c transcription by directly binding to conserved E-boxes of their promoter regions [122]. Thus, a reciprocal negative feedback loop involving ZEB1 and miRNA expression may tightly control tumor metastasis.

ZEB2 also constitutes a direct target of miRNAs. Ectopic ZEB2 rescues the suppressed cell migration and invasion mediated by miR-132, which is significantly down-regulated in NSCLC cell lines and clinical NSCLC tissue samples. [42]. MiR-138, miR-145, miR-215, and miR-598 also suppress lung cancer cell invasion and migration by targeting ZEB2 [49,55,83,92].

### 3.3. Twist TF-Related miRNAs

Twist, an EMT TF, is a target of miR-98, as their expression levels inversely correlates in clinical NSCLC tissue specimens. MiR-98 up-regulation suppresses cell invasion and migration by impeding Twist-induced EMT [56]. Bioinformatics analysis and luciferase-reporter assay revealed that miR-92b suppresses Twist to reduce NSCLC metastasis [50]. MiR-33a targets Twist family BHLH transcription factor 1 (Twist 1) to inhibit NSCLC invasion and metastasis in vitro and in vivo [43].

### 3.4. MiRNAs Modulate Other EMT-Associated Signaling Genes and Related Downstream Proteins 

Numerous EMT-associated signaling modulators are also regulated by miRNAs [123]. TGF-β, a well-known EMT activator, mediates cell proliferation, apoptosis, inflammation, tissue repair, and carcinogenesis [124]. Upon receptor binding, TGF-β induces receptor complex formation and activates downstream signals. TGF-β signaling includes Smad-dependent and Smad-independent pathways. In the former, Smad2/3/4 protein complex formation inhibits E-cadherin expression and induces fibronectin and matrix metalloprotease (MMP) expression, which regulates EMT processes. The latter involves RAS/RAF/ERK and PI3K/AKT signaling pathways, which are crucial for cell proliferation. [125,126]. Up-regulated TGF-β1 promotes lung adenocarcinoma invasion and metastasis via an EMT-associated mechanism [127]. Aberrant TGF-β up-regulation is critical to the development of targeted therapy resistance and disease progression in NSCLC [128,129,130,131]. MiRNAs target TGF-β pathway downstream factors to modulate EMT: miR-155 targets RhoA or Smad2/3, and miR-148a targets Rho-associated protein kinase I (ROCK1) [67,89,132]. MMPs digest most protein components in the ECM and participate in cell migration and invasion in physiological and pathological conditions, such as tissue remodeling and cancer cell progression [133].

NSCLC cells exhibit lower miR-148b expression than that in normal bronchial epithelial cells. A miR-148 mimic increased epithelial-associated E-cadherin and decreased mesenchymal-associated N-cadherin and vimentin expression. MiR-148b regulates ROCK1, a downstream TGF-β signaling factor, to inhibit cell proliferation and EMT, and increase sensitivity to radio-chemotherapy in NSCLC [93]. MiR-155-5p suppresses invasion and migration by targeting Smad2 [67]. MiR-215 represses NSCLC migration, invasion, and proliferation by directly targeting MMP-16 [134].

MiR-136 (miR-136-5p) inhibits lung cancer cell metastasis and EMT by directly targeting Smad2 and Smad3 [79]. Among a cohort of 1242 samples from the Gene Expression Omnibus and The Cancer Genome Atlas (TCGA) datasets, miR-136-5p was up-regulated in lung adenocarcinoma versus normal tissues. Bioinformatics analysis using Gene Ontology (GO), Kyoto Encyclopedia of Genes and Genomes (KEGG) and Protein Analysis Through Evolutionary Relationships (PANTHER) pathways demonstrated that claudin-18, sialophorin, and syndecan 2, which function in cell adhesion and focal adhesion, likely comprise miR-136-5p target genes [135]. Because of the intrinsic complexity and sophistication of tumor initiation and progression, miR-136-5p might exhibit disparate dysregulation and functions in various cancers [135].

In NSCLC and hepatocellular carcinoma, MET oncogene activated miR-221 and miR-222 by activating the c-JUN TF. These miRNAs suppress phosphatase and tensin homolog (PTEN) and tissue inhibitor of metalloproteinases 3 (TIMP3), and promote cellular invasion and migration by activating the Akt (Protein kinase B, PKB) murine thymoma viral oncogene homolog (AKT) pathway and metallopeptidase [69]. Somatic PTEN mutation occurs in 4–8% of NSCLC [136,137], whereas PTEN overexpression inhibits lung cancer cell invasion and metastasis by inhibiting integrin αVβ6 signaling [138]. In patients with NSCLC, decreased PTEN expression constitutes a poor prognosis factor [139]. PTEN is also a target of miR-19 and miR-26a to regulate EMT in NSCLC [44,57]. MiR-664 regulates tumorigenesis and malignant progression in lung cancer cell lines, with up-regulated miR-664 promoting cell invasion and migration by targeting PTEN [102].

Transmembrane serine protease 4, a membrane-anchored protease, mediates cell invasion and migration in a variety of cancers including lung cancer. This protein suppresses miR-205 (miR-205-5p) expression to promote EMT. In vivo, miR-205-5p expression inhibits cell growth, migration, and metastasis formation. MiR-205-5p directly targets integrin α5, a pro-invasive protein in NSCLC. Down-regulated integrin α5 expression in lung cancer cells completely abrogates cell migration, decreases the fibronectin adherence capacity, and reduces tumor growth in vivo [52].

Polycomb repressive complex 2 subunit (SUZ12) is involved in NSCLC tumor progression by promoting cell proliferation and metastasis [140]. MiR-489 targets SUZ12 to modulate EMT. MiR-489 down-regulation decreases E-cadherin protein level and increases N-cadherin and vimentin, which promote NSCLC invasion [85]. MiR-302b-3p inhibits NSCLC progression by targeting glucosaminyl (N-acetyl) transferase 3 (GCNT3), with E-cadherin, N-cadherin, vimentin, phosphorylated-extracellular-signal-regulated kinase, and cyclin D1 being downstream molecules of the miR-302b-3p/GCNT3 pathway [72]. GCNT3 expression patterns are associated with different cancer progression [141,142]. MiR-105 promotes NSCLC EMT by up-regulating myeloid cell leukemia 1 (Mcl-1) [62]. MiR-21 is highly expressed in the serum of patients with NSCLC, whereas its depletion reduces A549 cell proliferation, migration, and invasion by up-regulating programmed cell death protein 4 (PDCD4) expression [51]. MiR-455-5p promotes cell proliferation and invasion by targeting suppressor of cytokine signaling 3 (SOCO3) in NSCLC, wherein aberrant miR-455-5p expression is partially controlled by ERK signaling activation [80].

MiRNAs exhibit contradictory effects on EMT because their targets are cell-context dependent. MiR-590 (miR-590-3p) promotes A549 lung adenocarcinoma cell migration and invasion by targeting olfactomedin 4 (OLFM4), inhibiting tumor cell adhesion [94]. Conversely, miR-590 (miR-590-5p) down-regulation promotes NSCLC cell migration and invasion because it directly targets disintegrin and metalloproteinase 9 (ADAM9) [97]. MiR-590 (miR-590-5p) overexpression inhibits NSCLC cell proliferation and invasion by directly targeting Grb2-associated binder 1 (GAB1) [98]. The discrepancy of cell invasion and migration in miR-590 comes from the different sequences between miR-590-3p and miR-590-5p.

In summary, the EMT plays a crucial role in tumor invasion and metastasis, and it is also complex, multifunctional, and tightly regulated developmental program. Accumulating evidence suggests that microRNAs tightly regulate EMT in lung cancer cells. MicroRNAs act as pro- or anti-EMT through different targets and signal pathways, which regulates lung cancer invasion and metastasis.

## 4. Role of miRNAs in Different Metastasis Sites (Bone, Brain and Lymph Nodes) in Lung Cancer

**Figure 2:**
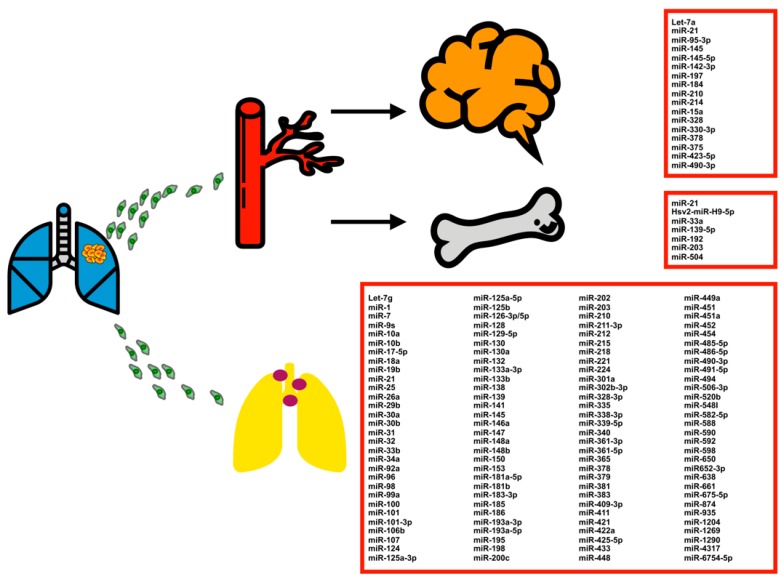
MiRNA expression profiling in NSCLC associated with brain, bone, and lymph node metastasis.

In addition to predicting patient survival and tumor relapse, patients with NSCLC with and without metastasis exhibit different miRNA profiles [143,144]. Numerous studies have investigated the association between miRNA expression profile and lung cancer metastatic sites [145] (Figure 2). 

### 4.1. Role of miRNAs in Lung Cancer Bone Metastasis

Bone metastasis occurs in approximately 15 to 30 percent of patients with lung cancer [146], representing one of the most deleterious clinical consequences [147]. However, the exact mechanism of bone metastasis remains unknown. The miRNAs associated with lung cancer bone metastasis are listed in Table 2.

A high-throughput sequencing study to explore the candidate bone metastasis-related miRNAs in lung adenocarcinoma generated two small RNA (corresponding to 18–30 nucleotides) libraries from the blood of patients with lung adenocarcinoma with and without bone metastasis. Expression profiling revealed 7 down-regulated and 21 up-regulated miRNAs in lung adenocarcinoma with bone metastasis. Bioinformatics analysis identified putative associated signaling pathways including MAPK, Wnt, and nuclear factor kappa light chain enhancer of activated B cells (NF-κB), along with pathways involving cytoskeletal proteins, angiogenesis factors, and MMP [148].

Moreover, 18 patients with NSCLC and vertebral column metastasis exhibited higher miR-21 expression levels than that in 20 patients with bone tuberculosis [149]. MiR-21 promotes cell proliferation and inhibits apoptosis in H2170 NSCLC cells through overexpression of cytochrome C oxidase assembly homolog 19 (COX19) [149], which affects COX subunit assembly by increasing COX activity. Reducing COX activity increases cytochrome C content, activating cell apoptosis signaling pathways and finally leading to apoptosis [150,151]. MiR-21 also mediates tumorigenesis and osteoclastogenesis by targeting PDCD4, which regulates osteoclastogenesis [152].

Some viruses regulate their own and/or host gene expression via aberrant miRNA expression [153,154]. Microarray analysis to compare miRNA expression in bone metastasis (*n* = 10) from lung cancer with that of primary lung cancers (*n* = 24) identified and validated a candidate viral miRNA, Hsv2-miR-H9-5p, encoded by herpes simplex virus type 2 latency-associated transcript [155]. Hsv2-miR-H9-5p expression is significantly higher in bone metastasis lesions than primary lung cancers. Hsv2-miR-H9-5p increases lung cancer cell migration and invasion in vitro by directly targeting suppressor of cytokine signaling 2 (SOCS2), inhibiting Jak2 kinase activity and Jak2-signal transducer and activator of transcription 3 (STAT3) binding [156]. SOCS2 expression is down-regulated in lung cancer [157].

MiR-139-5p serum levels from patients with lung adenocarcinoma and osteolytic bone metastasis are lower than those in patients with other organ metastasis. MiR-139-5p expression in mesenchymal stem cells (MSCs) significantly increases during osteogenic differentiation. Notch homolog 1, translocation-associated (Drosophila) (Notch1), a direct miR-139-5p target, exhibits significant down-regulation during MSC osteogenesis [159]. Tumor transfer of miR-192-enriched exosome-like vesicles to the endothelial compartment of the osseous milieu in vivo reduced bone metastases burden. MiR-192 overexpression confers anti-osseous metastatic activity in vivo and limits tumor-induced angiogenesis [160]. MiR-203/TGF-β/Smad2 expression represents an important tumor suppressor signaling pathway for bone metastasis in NSCLC, as patients with bone metastasis exhibited lower tumor tissue miR-203 expression than those without bone metastasis [161]. 

### 4.2. Role of miRNAs in Lung Cancer Brain Metastasis

Brain metastasis affects approximately 25% of patients with NSCLC during their lifetime [162]. However, no molecular biomarkers or effective indices are available to reduce brain metastasis risk. The mechanism of brain metastasis is also not completely clear owing to the limited available tissue specimens. Table 3 lists lung cancer brain metastasis-related miRNAs. 

MiRNA microarray-based comparison of expression profiles in five primary lung adenocarcinoma tumors versus three brain metastatic lung adenocarcinoma samples reveals obvious miR-145 down-regulation in brain metastatic samples, albeit no relationship between miR-145 and lymph node metastasis [163]. Among miRNAs from 527 patients with stage I NSCLC, miRNA microarray analysis identified 10 miRNAs associated with brain metastasis including miR-145 [164]. Promoter methylation-mediated miR-145-5p down-regulation promotes lung adenocarcinoma cell brain metastasis, whereas miR-145-5p expression reduces cancer cell migration [165].

MiR-21 is a target of STAT3 [179,180]. In patient-derived stem cell lines from lung-to-brain metastasis, miR-21 down-regulation attenuates brain metastasis-initiating cell self-renewal and migration comparably to STAT3 knockdown [166]. Compared to parental cells, miR-95-3p is down-regulated in brain metastasis cells generated through injection of lung adenocarcinoma cells into a left ventricle of nude mice. MiR-95-3p overexpression suppresses cell invasion, proliferation, and colony formation. Cyclin D1 was identified as a direct miR-95-3p target [167].

MiR-4317 is significantly down-regulated in tumor tissues compared with that in paired normal tissues, whereas patients with early stages and non-lymph node metastasis exhibit higher miR-4317 levels. MiR-4317 up-regulation significantly suppresses cell proliferation, colony formation, invasion, and migration. It also hampers NSCLC cell cycling by directly targeting fibroblast growth factor 9 (FGF9) and cyclin D2 (CCND2). In mouse xenograft model, miR-4317 suppresses tumor growth and brain and lung metastasis [178]. MiRNA microarray analysis identified miR-328 as related to brain metastasis by comparing samples from patients with (*n* = 7) and without (*n* = 8) brain metastasis. MiR-328 overexpression in A549 cells significantly promotes cell migration concomitant with protein kinase C alpha (PRKCA) up-regulation [171].

Overexpression of mir-423-5p, selected using microarray analysis of brain metastasis-related miRNAs and validated by quantitative PCR, promotes NSCLC cell colony formation, cell motility, migration, and invasion by direct targeting metastasis suppressor 1 (MTSS1). In clinical samples, lung adenocarcinoma tissues without brain metastasis exhibit positive staining for MTSS1 expression [176]. Microarray analysis between patients with and without brain metastasis revealed that a three-miRNA (including miR-210, miR-214, and miR-15a) signature predicts the brain metastasis of patients with lung adenocarcinoma with high sensitivity and specificity [170]. 

Recently, increasing evidence revealed that exosomes play important roles in the tumor microenvironment and the mechanism of malignant tumor metastasis. Exosomes, consist of a phospholipid bilayer, which is composed mainly of proteins, lipids, carbohydrates, and nucleic acids [181,182]. Exosome carries miRNAs, termed “exomiRs”, to acceptor cells to promote non-adjacent intercellular communication, which involves in cell differentiation, immune response, antigen presentation, and cell invasion/migration [183,184,185]. The transfer of exosomal miRNA can modulate gene expression in acceptor cancer cells to facilitate metastasizing cancer cell settlement in pre-metastatic organs, suggesting these exosomal miRNAs prepare the pre-metastatic niche [186]. 

Astrocytes oppose brain metastasis via exosome-delivered miR-142-3p, which directly binds to the suppressing transient receptor potential ankyrin-1 (TRPA1) 3′UTR. TRPA1 also directly targets the FGF receptor 2 C-terminal proline-rich motif, thereby constitutively activating the receptor and increasing lung adenocarcinoma progression and metastasis [168]. Transferring miR-142-3p from astrocytes to lung cancer cells suppresses TRPA1 in the latter, promoting brain metastasis. MiR-184 and miR-197 are also overexpressed in patients carrying EGFR mutation with brain metastasis; their expression level may serve to stratify the brain metastasis risk in this subpopulation [169].

### 4.3. Role of miRNAs in Lung Cancer Lymph Node Metastasis

Lymphatic metastasis comprises an important mechanism in tumor spreading in addition to metastasis via blood vessels. The primary epithelial cancer cells enter into the lymphatic drainage system and spread to local or distal lymph nodes after penetrating the basement membrane [187]. For patients with early stage lung cancer, lymphatic invasion or lymph node involvement represents a key prognostic factor. Regional lymph node status is important for lung cancer staging and treatment planning [188]. However, traditional image examination (chest CT) sensitivity is poor. Micro-metastasis or occult lymph node metastasis is still found in approximately 20% of early stage (T1/T2) lung cancer tumors [189,190]. Positron emission tomography (PET) scanning and endobronchial ultrasound-guided transbronchial needle aspiration can decrease the high false-negative rate and provide greater sensitivity and specificity for mediastinal lymph node assessment [191,192,193]. The identification of molecular biomarkers expressed in tumor tissue or patient serum is helpful to predict lymph node metastasis. Table 4 lists the different miRNAs related to lymph node metastasis.

The role of miR-200c in lung cancers is controversial. MiR-200c inhibits NSCLC cells invasion and migration, and expression of the miR-200c targets USP25 in NSCLC correlates with clinical stage and lymphatic node metastasis [115]. Lower miR-200c expression also significantly correlates with poor differentiation grade, lymph node metastasis, and lower E-cadherin expression [115,275]. However, higher tumor miR-200c expression was reportedly associated with poor survival in patients with NSCLC [209,276].

MiR-125a-3p/5p is down-regulated in NSCLC tissues compared with adjacent normal lung tissues. However, the relationship with metastasis differs between the two mature miRNAs, which are derived from the 3′ and 5′ ends of pre-miR-125a. Patients with low miR-125a-3p and high miR-125a-5p expression exhibit increased lymph node metastasis [235]. A similar correlation is reported between miR-125a-3p and lymph node metastasis [236]. Alternatively, miR-125a-5p overexpression inhibits lung adenocarcinoma cell proliferation and induces cell apoptosis by targeting neural precursor cell expressed, developmentally down-regulated 9 (NEDD9). MiR-125a-5p expression negatively correlates with lymph node metastasis [237]. In turn, miR-125b exhibits tumor suppressor function by targeting MMP-13 to inhibit cell invasion. Decreasing miR-125b in tumor tissues correlates with lymph node metastasis [238].

MiR-130 also plays a controversial role in NSCLC. MiR-130 is significantly down-regulated in NSCLC tumor tissues and cell lines. High miR-130 expression inversely correlates with lymph node metastasis and late stages. MiR-130 up-regulation significantly suppresses NSCLC cell growth and enhances cell apoptosis by directly targeting PTEN [243]. MiR-130 family consists of miR-130a and miR-130b, and they have nearly identical sequences, although miR-130a and miR-130b come from chromosome 11 and chromosome 22, respectively. MiR-130a functions as a proangiogenic miRNA and antagonizes the inhibitory effect of growth arrest homeobox transcription factor and homeobox A5 (HoxA5) on endothelial cell proliferation, migration, and tube formation [338]. MiR-130a is also overexpressed in NSCLC tissues, with higher expression being strongly associated with lymph node metastasis and poor prognosis [244]. Further studies are thus needed to clarify the role of miR-130 in NSCLC.

Serum miRNA levels also serve as biomarkers of NSCLC metastasis or prognosis [209,213]. High serum miR-21 correlates with advanced stages and lymph node metastasis. MiR-21 promotes cell proliferation, metastasis, and chemo-radio-resistance in NSCLC cells by targeting PTEN [211]. High serum miR-19a and miR-19b also significantly correlate with tumor-node-metastasis (TNM) stage and lymph node metastasis [207,208]. Patients with NSCLC exhibit significantly increased serum miR-494 levels compared with those in healthy controls, with the levels markedly decreasing when patients receive effective therapy. MiR-494 up-regulation in serum or tumor tissues significantly associates with higher incidence of lymph node metastasis, advanced clinical stage, and higher histological grade [317,318]. MiR-210, miR-421, and miR-411 levels in tumor tissues or serum of patients with lung cancer significantly positively correlate with lymph node metastasis and poor prognosis [279,280,300,301]. Patients with NSCLC exhibit lower serum miR-138 than that of healthy controls. Low miR-138 expression correlates with positive lymph node metastasis and poor prognosis [248]. MiR-138 suppresses NSCLC proliferation, metastasis, and autophagy by targeting sirtuin 1 (Sirt1) [249]. MiR-138 also targets Yes-associated protein 1 (YAP1) [250].

MiRNAs are detected in sputum and plasma [313]. Between lung cancer tissues with adjacent non-cancerous specimens, the former show lower miR-486-5p expression, with the reduced expression being associated with advanced clinical stage and lymph node metastasis of NSCLC [312,313,314]. In vitro, miR-486-5p down-regulation promotes tumor progression and metastasis by targeting Rho GTPase-activating protein 5 (ARHGAP5). MiR-486-5p expression in sputum and plasma specimens could provide a diagnostic approach for early lung cancer detection [314]. Moreover, miR-486-5p up-regulation in cancer cells reduces expression of Pim-1, a direct target. Pim-1 kinase, a proto-oncogene, is overexpressed in 66.2% of lung tumor tissues by immunohistochemical staining. Pim-1 expression is significantly higher in NSCLC tissues than in adjacent normal tissues [315].

Meta-analysis from the TCGA database demonstrated that lower miR-133a-3p correlates with negative lymph node metastasis and might act as a tumor suppressor [245]. In lung adenocarcinoma, miR-452-5p expression is obviously lower than that in adjacent normal tissues, and negatively correlates with lymph node metastasis and TNM stage [310]. MiR-145-5p shows similar findings among 125 paired NSCLC tissues and the TCGA database, indicating that both miRNAs function as tumor suppressors [255]. Among 372 NSCLC and 42 adjacent normal lung tissues from the Gene Expression Omnibus dataset, miR-101-3p showed higher expression in normal than NSCLC tumor tissues. Low miR-101-3p expression significantly correlated with lymph node metastasis and shorter OS [228].

Gene promoter methylation generally results in down-regulation of gene expression. Aberrant miR-200c promoter methylation obviously negatively correlates with miR-200c expression and is associated with lymph node metastasis and poor clinical outcome [275]. Histone methylation-mediated (H3K27me3) miR-139 silencing enhances NSCLC invasive and metastatic phenotype, with down-regulated miR-139 expression being significantly associated with lymph node metastasis and tumor invasiveness [251].

MiRNAs are also influenced by other non-coding RNAs, such as long noncoding RNA (lncRNA), which are over 200 nucleotides in length and exert their effects in the form of RNA. The lncRNA LINC00978 promotes cell proliferation and invasion in NSCLC by inhibiting miR-6754-5p [337]. HOXD-AS1 promotes NSCLC migration and invasion by regulating the miR-133b/MMP9 axis, with miR-133b being a direct target of HOXD-AS1 in NSCLC [247]. LncRNA NNT-AS1 promotes lung cancer cell proliferation and invasion by regulating miR-129-5p [242]. LncRNA SNHG15 promotes NSCLC proliferation and migration by targeting miR-211-3p, with high SNHG15 expression levels correlating with tumor size and lymph node metastasis [281]. LncRNA HNF1A-AS1 promotes cell proliferation and invasion by directly targeting miR-17-5p in NSCLC [205]. HOXA11-AS acts as a ceRNA to regulate TF Sp1 expression via sponging miR-124 [234]. NEAT1 promotes proliferation and invasion by targeting miR-181a-5p [264]. LncRNA XLOC_008466 functions as an oncogene in NSCLC by regulating the miR-874-MMP2/XIAP axis. XLOC_008466 up-regulation in patients with NSCLC was related to lymph node metastasis and TNM stage [331]. LncRNA SNHG1 overexpression promotes NSCLC progression by inhibiting miR-101-3p and activating the Wnt/β-catenin signaling pathway [229]. More examples can be found in recent reviews [339,340].

## 5. Potential of miRNA as a Therapeutic Target and Tool in Patients with Lung Cancer

Increasing evidence demonstrates that miRNAs play pivotal roles in lung cancer invasion and metastasis. Studies using miRNA profiling to predict prognosis and clinical treatment response indicate that miRNA expression profiles can predict patient cancer relapse and clinical outcome in NSCLC [143]. The European Lung Cancer Working Party (ELCWP) prospective study, initiated to identify a miRNA-based signature for treatment response and survival for NSCLC treated with cisplatin and vinorelbine, revealed that a four-miRNA signature (miR-200c, miR-424, miR-29c, and miR-124) could predict treatment response of first-line cisplatin and vinorelbine and act as a prognostic factor in patients with NSCLC [341]. The combination of a plasma immune-related microRNA-signature classifier and immunohistochemical stain of programmed death-ligand 1 in tumor specimens could predict poor treatment response and OS in patients with NSCLC treated with immune-checkpoint inhibitors [342].

For predicting disease prognosis, gain- and loss-of-function studies of miRNAs have provided a rationale and innovative insight toward precision medicine by targeting miRNA to prevent tumor progression or spreading of cancer cells, because miRNAs can stably modulate gene networks [343]. Possible approaches include: (i) miRNA-based treatment (Direct strategy). Introduction of synthetic miRNA analogs (miR mimics) to mimic tumor suppressor miRNAs that are down-regulated in cancer cells, or antisense oligonucleotides (known as anti-miRs or antagomiR) to silence oncogenic/metastasis-promoting miRNAs [344,345]. Although ectopic expression of synthetic miRNAs mimics was accomplished in vitro, there was little in vivo data using miRNA mimics delivered by intravenous injection. The expression of miRNAs is also restored by inserting genes coding for miRNAs into viral constructs, such as the adenovirus-associated vectors (AAV) [346,347,348]. These vectors do not integrate into the genome and have high efficiency of transduction. Kota and colleagues cloned miR-26a into an AAV vector and viral particles were tested in a mouse model of liver cancer. Systemic administration of miR-26a results in inhibition of cancer cell proliferation, induction of tumor-specific apoptosis, and dramatic protection from disease progression without toxicity [348]. Besides, miRNA-based treatment involves various strategies, including miR-mask and miRNA sponges which interrupt the interaction between target and miRNAs [346]. (ii) Induction of miRNA expression (Indirect strategy). Some drugs were developed to modulate the expression of miRNAs by regulating activation or repression of upstream transcription factors. By screening for more than 1000 small molecular compounds, diazobenzene 1 promoted transcription of miR-21 and produced a 250% increase of miR-21 relative to the untreated cells [349]. 

A critical challenge of targeted miRNA therapy is how to introduce the synthetic oligonucleotide or miRNA mimic into the cancer cells. Viral and non-viral vectors comprise commonly used vectors for miRNA delivery [350]. However, viral vector introduction into the host system can trigger an immune response [351]. Systemic treatment with miR-10b antagomir, a 2’-O-methyl-group (OMe)-modified, cholesterol-conjugated antisense miR, and miR-34a mixed with atelocollagen could suppress breast and colon cancer metastasis, respectively, in animal studies [352,353]. Systemic delivery of miR-34a into experimental lung metastasis of murine B16F10 melanoma using a liposome-polycation-hyaluronic acid nanoparticle formulation modified with tumor-targeting single chain antibody fragment (scFv) reduces tumor load in the lung [354].

Several pre-clinical and clinical trials of miRNA as targeting therapy for lung cancer are ongoing (Table 5). Let-7 suppresses lung tumor via KRAS in vivo, and exogenous lentivirus-mediated let-7 delivery significantly reduces the tumor burden in mouse models of NSCLC [355]. Systemic let-7 or miR-34a delivery by injection of neutral lipid emulsion also significantly attenuates tumor burden in the KRAS autochthonous NSCLC mouse model [272,356]. For EGFR mutant NSCLC, combinatorial treatment with let-7b and miR-34a provides synergistic treatment effect with erlotinib, an EGFR tyrosine kinase inhibitor, to suppress NSCLC proliferation [357]. Plasmid-mediated miR-126 plasmid inhibits A549 cell proliferation in vitro and inhibits tumor growth in vivo by increasing expression of EFG–like domain 7 [358]. MiR-145 inhibits NSCLC proliferation by directly targeting c-Myc in vitro [359]. Cationic polyurethane-short branch polyethylenimine (PEI) -mediated delivery of miR-145 inhibits xenograft tumor growth, EMT, and metastasis, and prolongs the survival times of a lung adenocarcinoma mouse model [360]. Efficient systemic delivery of miR-133-b and miR-29 by cationic lipoplexes inhibits tumor growth in vitro and in vivo [361,362]. The first miRNA mimic-based therapy, MRX34 (Mirna Therapeutics Inc., Austin, TX, USA.), a liposomal miR-34 mimic, entered phase I clinical trial of liver cancer therapy in 2013 [363], demonstrating acceptable safety and antitumor activity of MRX34 in a subset of patients with refractory advanced solid tumors [364], along with positive results of lung cancer in vitro and in animal studies [356,365]. However, the further phase I/II clinical trials (ClinicalTrials.gov identifiers: NCT01829971, NCT02862145) were terminated or withdrawn because the suitability of associated serious immune-related adverse events for clinical application was questioned. 

MesomiR 1 (NCT02369198), a first-in-man, phase I clinical trial, enrolled patients with NSCLC and malignant pleural mesothelioma to assess the safety and activity of TargomiRs as the second and third line of treatment [367]. TargomiRs (TargomiRs; EnGeneIC Ltd., Sydney, Australia) comprise minicells loaded with a miR-16-based mimic, which acts as an anti-EGFR specific antibody. The MesomiR 1 study intended to specifically deliver miR-16 to suppress tumor development, as the family of this miRNA is associated with tumor suppression in several cancers. The clinical trial demonstrated the acceptable safety profile in patients with malignant pleural mesothelioma [366]. Future research is necessary to address clinical treatment efficacy.

Argonaute-2 (AGO2) mediates post-transcriptional gene silencing, as an essential component of the RNA-induced silencing complex (RISC). After miRNA assembles into RISC, the activation complex silences and degrades the target mRNA transcripts [368]. However, when a double-strand RNA loads into AGO2, the AGO2-bound RNAs can activate transcription in the nucleus, paradoxically increasing mRNA expression [343,369]. According to this mechanism, a phase 1 trial (NCT02716012) of hepatocellular carcinoma was launched for a small activating RNA (saRNA) drug to increase CCAAT/enhancer-binding protein α (C/EBPα) expression. The dsp21 (saRNA) is designed to activate p21WAF1/CIP1 gene expression, and it inhibits cell proliferation, and induces apoptosis in lung cancer H441 and A549 cells [370,371]. More importantly, dsp21 increased the chemo-sensitivity to cisplatin of lung cancer cells in vitro and in vivo [371]. This synergistic effect of saRNA and chemotherapy may provide a reasonable concept for developing a treatment strategy in lung cancers. In addition to chemotherapy, aberrant activity of oncogenic pathways are the characteristics of carcinogenesis, it is worth noting that the combined treatment strategy of miRNA and saRNA to concurrently silence and activate opposing pathways of cancer. The combination strategies may develop more potent precision therapies of cancers [372].

There are several advantages of miRNA-based therapeutic in lung cancer over other treatment strategies, such as targeting growth factor receptors or enzymatic proteins. MiRNA-based therapies have emerged as promising therapeutic tools for cancer management due to highly specific in tissues and tumors. In addition, the advantage of using miRNA approaches is based on the ability to concurrently target multiple effectors of pathways involved in cell differentiation, proliferation, and survival. Therefore, miRNAs therapies have extremely efficiencies in regulating distinct biological cell processes relevant to malignant cell homeostasis [346,373]. The ability of miRNAs to regulate multiple genes in a molecular pathway makes them excellent candidates for novel molecular-targeting treatments.

However, there are still some problems with miRNA therapies. Therapeutic miRNAs is difficult to cross through cell membranes resulting in poor cellular uptake of oligonucleotides because of the size and negative charge of miRNAs. In addition, delivering a therapeutic miRNA to the associated target tissues also challenges. MiRNAs are relatively unstable and reduce their half-life substantially in the blood circulation due to subject to rapid degradation by RNases [272]. It is an obstacle to achieve a sufficient amount of synthetic oligonucleotides to sustain the inhibitory effect [346,374,375]. Finally, the potential off-target effects of miRNA therapeutics are major concerns because of concurrent regulation of many genes. In addition, recent reports revealed the toxicity related to miRNA therapies [376]. These pharmacokinetic and pharmacological drawbacks of RNA-based therapeutics, such as off-targeting, low serum stability, and innate immune responses, require more research.

Although miRNA replacement therapy remains challenging with numerous problems needing to be resolved, several clinical trials with miRNA mimics have already been initiated. By developing more specific carriers and expression models, regulation of miRNA function will likely become more specific and effective for cancers. Cancer therapy through miRNA regulation may thus engender a new era for cancer patients in the near future.

## 6. Conclusions

Lung cancer remains a major cause of cancer-associated deaths globally. The aggressive behavior of lung cancer involved in invasion and migration caused disease rapid progression despite standard treatment. MiRNAs regulate multiple genes and different signal pathways. Increasing studies suggested that miRNAs reveal discrete expression patterns in lung cancers. Dysregulation of miRNA expression regulates EMT and cancer metastasis by targeting various genes. Different miRNA expression in tumor tissues or sera is associated with different metastatic sites. These miRNA profiles also correlate with prognosis and clinical treatment response in lung cancers and could be potential targets of lung cancer treatment. More research on miRNA targeted therapies is necessary to increase the target specificity and potency and decrease the off-target effects and toxicity. Exploring miRNA-targeted therapy may establish a new spectrum of lung cancer treatments.

## Figures and Tables

**Table 1:** Epithelial-to-mesenchymal transition-related transcription factors and associated microRNAs in Non-Small Cell Lung Cancer (NSCLC).

Epithelial-to-Mesenchymal Transition-Related Transcription Factors						
**Snail**	**Slug**	**ZEB1**	**ZEB2**	**Twist**	**Other (miRNA/Related Gene or Target)**	
miR-22 [36]	miR-1 [40]	miR-33b [41]	miR-132 [42]	miR-33a [43]	miR-19/PTEN [44]	miR-204/SIX1 [45]
miR-30 [37,38]	miR-124 [46,47]	miR-34a [48]	miR-138 [49]	miR-92b [50]	miR-21/Pdcd4 [51]	miR-205-5p/Integrin α5 [52]
miR-34 [33]	miR-137 [53]	miR-101 [54]	miR-145 [55]	miR-98 [56]	miR-26a/PTEN [57]	miR-205-5p/Smad4 [58]
miR-126 [34]	miR-218 [59]	miR-124 [60]	miR-154 [61]	miR-381 [39]	miR-105/Mcl-1 [62]	miR-206/MET [63]
miR-346 [35]	miR-452 [64]	miR-144 [65,66]	miR-155-5p [67]		miR-124/STAT3 [68]	miR-221&222/PTEN, TIMP3 [69]
miR-381 [39]		miR-155-5p [67]	miR-200c [70]		miR-133/FOXQ1 [71]	miR-302b-3p/GCNT3 [72]
		miR-181 [73]	miR-205-5p [74]		miR-135b/LZTS1, Hippo pathway [75]	miR-361-3p/SH2B1 [76]
		miR-199b [77]	miR-203 [78]		miR-136/Smad2/3 [79]	miR-455-5p/SOCO3 [80]
		miR-200s [81,82]	miR-215 [83]		miR-145/N-cadherin [84]	miR-489/SUL12 [85]
		miR-205-5p [74]	miR-218 [59]		miR-145/MTDH [86]	miR-497/MTDH [86]
		miR-216a [87]	miR-338-3p [88]		miR-148a/ROCK1 [89]	miR-544a/cadherina 1 [90]
		miR-455-3p [91]	miR-598 [92]		miR-148b/ROCK1 [93]	miR-590-3p/OLFM4 [94]
		miR-1199-5p [95]			miR-150/FOXO4 [96]	miR-590-5p/ADAM9 [97]
					miR-155-5p/Smad2/3 [67]	miR-590-5p/GAB1 [98]
					miR-183/MTA1 [99]	miR-598/Derlin 1 [100]
					miR-191/HIF-2α [101]	miR-664/AKT [102]
					miR-195/MYB [103]	miR-876-5p/BMP-4 [104]
					miR-196a/HOXA5 [105]	miR-1260b/PTPRK [106]

PTEN: phosphatase and tensin homolog; PDCD4: programmed cell death protein 4; TWIST1: twist family BHLH transcription factor 1; Mcl-1: myeloid cell leukemia 1; STAT3: signal transducer and activator of transcription 3; FOXQ1: forkhead box protein Q1; MTDH: metadherin; ROCK1: Rho-associated coiled-coil containing protein kinase; FOXO4: forkhead box protein O4; MTA1: metastasis-associated protein 1; HIF-2α: hypoxia-inducible factor 2-alpha; MYB: myeloblastosis; HOXA5: homeobox A5; SIX1: sineoculis homeobox homolog 1; MET: hepatocyte growth factor receptor; TIMP3: tissue inhibitor of metalloproteinase 3; GCNT3: glucosaminyl (N-acetyl) transferase 3, mucin type; SH2B1: Src homology 2B (SH2B) family member 1; SOCO3: suppressor of cytokine signaling 3; SUL12: polycomb repressive complex 2 subunit; OLFM4: olfactomedin 4; ADAM9: a disintegrin and metalloproteinase 9; GAB1: Grb2-associated binder 1; AKT: protein kinase B; BMP-4: bone morphogenetic protein 4; PTPRK: protein tyrosine phosphatase, receptor type K.

**Table 2:** Bone metastasis-related miRNAs in NSCLC.

miRNA	Direct or Related Target	Tumor Suppressor/Oncogene	Tissue	Effect	Author/Reference
miR-21	COX19	Oncogene	Metastatic bone tissue	Promoted cell proliferation, inhibited apoptosis	Guo et al. [149]
miR-21	PDCD4	Oncogene	TCGA database	Promoted osteoclastogenesis and tumorigenesis. High miR-21 correlated with poor prognosis according to TCGA database.	Xu et al. [152]
Hsv2-miR-H9-5p	SOCS2	Oncogene	Lung tumors	Increased cell survival, migration, and invasion	Wang et al. [155]
miR-33a	PTHrP	Suppressor	Lung cancer cell lines (A549, H1299, and BEAS-2B)	Reduced the stimulatory effect of A549 on the production of osteoclastogenesis activator RANKL and M-CSF on osteoblasts, and increased the production of osteoprotegerin.	Kuo et al. [158]
miR-139-5p	Notch1	Suppressor	Serum	MiR-139-5p expression was increased during MSC differentiation toward osteoblasts and positively regulated osteogenic differentiation.	Xu et al. [159]
miR-192	ICAM-1 and PTPRJ	Suppressor	In vivo (mice)	Decreased tumor-induced angiogenesis	Valencia et al. [160]
miR-203	TGF-β/SMAD2	Suppressor	Tumor tissues	Suppressed cell proliferation and migration, induced apoptosis; repressed TGF-β/Smad2	Wei et al. [161]

COX19: cytochrome C oxidase assembly homolog 19; PDCD4: programmed cell death 4; SOCS2: suppressor of cytokine signaling 2; PTHrP: parathyroid hormone-related protein; Notch1: Notch homolog 1, translocation-associated (Drosophila); RANKL: receptor activator of nuclear factor κ-B ligand; M-CSF: macrophage colony-stimulating factor; MSC: mesenchymal stem cell.

**Table 3:** Brain metastasis-related microRNAs in NSCLC.

miRNA	Direct or Related Target	Tumor Suppressor/Oncogene	Tissue	Effect	Author/Reference
miR-21	SPRY2, TIMP3, CDKN1A, SERPINB5 and PTEN.	Oncogene	In vivo	Promoted brain metastasis-initiating cell (BMIC) self-renewal and proliferation.	Singh et al. [166]
miR-95-3p	Cyclin D1	Suppressor	In vivo	Inhibited cell invasion, proliferation, and colony formation.	Hwang et al. [167]
miR-145		Suppressor	Brain and lung tumors	Inhibited cell proliferation	Zhao et al. [163]
miR-145-5p	TPD52	Suppressor	Brain and lung tumors	Inhibited cell invasion and migration. Restrained brain orthotopic tumor engraftment in vivo.	Donzelli et al. [165]
miR-142-3p	TRPA1	Suppressor	TCGA data	Suppressed NSCLC progression	Berrout et al. [168]
miR-184, miR-197			EGFR-mutant lung tumors		Remon et al. [169]
miR-15a, miR-210, miR-214			Lung tumor	The Forest model of the three-miRNA signature could be used to predict brain metastasis of lung adenocarcinoma patients	Zhao et al. [170]
miR-328	PRKCA	Oncogene	Brain and lung tumors	Increased cell migration. Up-regulated PRKCA	Arora et al. [171]
miR-330-3p	GRIA3	Oncogene	Lung tumors	Promoted cell growth, invasion, and migration. Up-regulated total DNA methylation. Radiation-resistance	Wei et al. [172] Jiang et al. [173]
miR-375	VEGF and MMP-9	Suppressor	Brain and lung tumors		Chen et al. [174]
miR-378	MMP-2, MMP-9 and VEGF	Oncogene	Brain and lung tumors	Promoted cell migration, invasion, and tumor angiogenesis.	Chen et al. [175]
miR-423-5p	MTSS1	Oncogene	Lung tumors	Promoted NSCLC cell colony formation, cell motility, invasion, and migration.	Sun et al. [176]
miR-490-3p	PCBP1	Oncogene	Brain tissues	Promoted cell proliferation, invasion, and migration.	Li et al. [177]
miR-590	ADAM9	Suppressor	Lung tumors	Suppressed tumorigenesis and invasion.	Wang et al. [97]
miR-4317	FGF9 and CCND2	Suppressor	Lung tumors	Inhibited proliferation, colony formation, migration, and invasion, and hampered cycling	He et al. [178]

NTSS1: metastasis suppressor 1; TPD52: tumor protein D52; TRPA1: transient receptor potential ankyrin 1; GRIA3: glutamate receptor, ionotropic, AMPA 3; MTSS1: metastasis suppressor protein 1; PCBP1: poly r(C)-binding protein 1; ADAM9: a disintegrin and metalloproteinase 9; FGF9: fibroblast growth factor 9; CCND2: cyclin D2; PRKCA: protein kinase C-α; MMP: matrix metalloprotease; TCGA: The Cancer Genome Atlas.

**Table 4:** Lymph node metastasis-related microRNAs in NSCLC.

miRNA	Direct or Related Target	Tumor Suppressor/Oncogene	Tissue	Effect	Author/Reference
Let-7g	HMGA2, ERCC6 and MAP3K3 *	Suppressor	Lung tumor	The combination of Let-7g and miR-21 profiling and KRAS mutational status may be considered a useful biomarker for clinical management of NSCLC patients.	Capodanno et al. [194]
miR-1	PIK3CA	Suppressor	Lung tumors	Low expression of miR-1 and overexpression of PIK3CA in NSCLC tissues may be useful predictors of lymph node metastasis and postoperative recurrence in patients with NSCLC.	Zhao et al. [195]
miR-7	Bcl-2	Suppressor	Lung tumor	Overexpressed CDR1as in NSCLC functioned to promote tumor progression via miR-7 signals. Up-regulated miR-7 increased the sensitivity of lung adenocarcinoma cells to CDDP by inducing apoptosis.	Zhang et al. [196], Cheng et al. [197]
miR-9s		Oncogene	Lung tumors	Involved in NSCLC progression and could serve as a promising biomarker.	Muraoka et al. [198], Xu et al. [199]
miR-10a	PTEN	Oncogene	Lung tumors	Promoted NSCLC cell proliferation, migration, and invasion.	Yu et al. [200]
miR-10b	E-cadherin	Oncogene	Lunt tumors PBMC	E-cadherin mRNA and protein were overexpressed in miR-10b-suppressed cells compared with controls. MiR-10b expression in PBMCs had predictive value for tumor response to chemotherapy and prognosis for advanced NSCLC patients.	Zhang et al. [201], Yang et al. [202], Yang et al. [203], Li et al. [204]
miR-17-5p		Oncogene	Lung tumors	Increased cell proliferation. LncRNA HNF1A-AS1 promoted cell proliferation and invasion by directly targeting miR-17-5p in NSCLC.	Zhang et al. [205]
miR-18a		Oncogene	Lung tumors	Correlated with stage, lymph node metastasis, and radio-resistance.	Shen et al. [206]
miR-19a/b		Oncogene	Lunt tumors, Serum	MiR-19b is a potential biomarker for the prediction of survival and response to chemotherapy in NSCLC.	Lin et al. [207], Wu et al. [208]
miR-21	PTEN	Oncogene	Lung tumors, Serum	Reduced radio-sensitivity in vitro. Promoted cell proliferation and cell cycle progression. High serum level was associated with poor prognosis.	Liu et al. [209], Wang et al. [210], Liu et al. [211], Wang et al. [212], Tian et al. [213]
miR-25		Oncogene	Lung adenocarcinoma tissues	Positive correlation with lymph node metastasis, stage, and EGFR mutations.	Xu et al. [214]
miR-26a	PTEN	Oncogene	Lung tumors	Enhanced lung cancer cell migration and invasion abilities. Up-regulated β-catenin, MMP-2, Twist, and VEGF.	Liu et al. [57]
miR-29b	MMP2	Suppressor	Lung tumor	Suppressed migration and invasion.	Wang et al. [215]
miR-30a	BCL11A	Suppressor	Lung tumors	A potential diagnostic and prognostic biomarker.	Jiang et al. [216]
miR-30b	EGFR Cthrc1	Suppressor	Lung tumors	Inhibited proliferation, migration, and invasion, induced apoptosis, and enhanced sensitivity of NSCLC cells to EGFR-TKIs.	Qi et al. [217], Chen et al. [218]
miR-31	CDK5, PTEN, p70S6K, ERK/MAPK, and PI3K/AKT ^#^	Oncogene	Lung tumors	Promoted cell proliferation, invasion, and migration.	Meng et al. [219]
miR-32		Suppressor	Lung tumors	Inversely correlated with tumor stage, lymph node metastasis, and OS.	Bai et al. [220]
miR-33b	ZEB1	Suppressor	Lung tumors	Inhibited cell growth, invasion, and EMT by suppressing Wnt/β-catenin/ZEB1 signaling.	Qu et al. [41]
miR-34a		Suppressor	Lung tumors and plasma	Plasma miR-34a negatively predicted lymph node metastasis. Lower miR-34a was correlated with longer survival.	Zhao et al. [221]
miR-92a	PTEN	Oncogene	Lung tumors	Promoted cell growth, metastasis, and chemo-resistance.	Ren et al. [222]
miR-96	FOXO3	Oncogene	Lung tumors	Promoted cell invasion and inhibited apoptosis.	Li et al. [223]
miR-98		Suppressor	Serum	Low serum miR-98 was positively correlated with advanced TNM stage, lymph node metastasis, and unfavorable OS.	Wang et al. [224]
miR-99a	mTOR	Suppressor	Lung tumors	Inversely correlated with lymph node metastasis.	Gu et al. [225]
miR-100	PLK1	Suppressor	Lung tumors	Inhibited cell proliferation and caused G2/M cell cycle arrest	Liu et al. [226]
miR-101	Mcl-1 ZEB1	Suppressor	Lung tumors	Inhibited cell proliferation, invasion, and migration.	Luo et al. [227], Han et al. [54]
miR-101-3p	SOX9	Suppressor	Lung tumors	LncRNA SNHG1 contributed to the progression of NSCLC through inhibition of miR-101-3p and activation of the Wnt/β-catenin signaling pathway.	Lu et al. [228], Cui et al. [229]
miR-106b		Oncogene	Lung tumor	Overexpression of miRNA-106b was strongly associated with lymph node metastasis and poor prognosis.	Li et al. [230]
miR-107	BNDF	Suppressor	Lung tumors	miR-107 significantly inversely correlated with tumor progression and decreased survival in patients with NSCLC.	Zhong et al. [231], Xia et al. [232]
miR-124	SOX8, STAT3	Suppressor	Lung tumors	Inhibited cell proliferation and induced apoptosis. HOXA11-AS acted as a competing endogenous RNA to regulate transcriptional factor Sp1 expression by sponging miR-124.	Xie et al. [233], Li et al. [68], Yu et al. [234]
miR-125a-3p	IGF2, CCL4	Suppressor	Lung tumors	Suppressed cell invasion and migration. Inversely correlated with lymph node metastasis.	Jiang et al. [235], Hou et al. [236]
miR-125a-5p	NEDD9	Uncertain	Lung tumors	The effects on cell invasion and migration and the relationship between miR-125a-5p and lymph node metastasis were controversial in lung cancers.	Jiang et al. [235] Zheng et al. [237]
miR-125b	MMP13	Suppressor	Lung tumors	Inhibited cell invasion in vitro and in vivo.	Yu et al. [238]
miR-126-3p/5p	44 co-targets ^¶^	Suppressor	Lung tumors,	Lower expression of miRNA-126-3p and -5p was indicative of vascular invasion, lymph node spread, and an advanced TNM stage of lung adenocarcinoma.	Chen et al. [239], Chen et al. [240]
miR-128	VEGF-C	Suppressor	Lung tumors	Inhibited cell proliferation, colony formation, invasion, and migration.	Hu et al. [241]
miR-129-5p		Suppressor	Lung tumor	LncRNA NNT-AS1 exerted functions in NSCLC by altering NNT-AS1/miR-129-5p axis	Shen et al. [242]
miR-130	PTEN	Suppressor	Lung tumor	Inhibited NSCLC cell growth and increased cell apoptosis.	Ye et al. [243]
miR-130a		Oncogene	Lung tumor	Overexpressed in NSCLC tissue and correlated with lymph node spreading.	Wang et al. [244]
miR-132	ZEB2	Suppressor	Lung tumor	Inhibited cell proliferation, invasion, and migration, and decreased apoptosis	You et al. [42]
miR-133a-3p		Suppressor	Lung tumor (TCGA)	Associated with longer survival time and negative lymph node metastasis.	Yang et al. [245]
miR-133b	EGFR	Suppressor	Lung tumor	Inhibited cell invasion, induced apoptosis, and enhanced sensitivity to gefitinib. HOXD-AS1 directly targeted miR-133b to promote cell migration and invasion.	Liu et al. [246], Xia et al. [247], Chen et al. [240]
miR-138	PDK1, Sirt1, YAP1	Suppressor	Lung tumor	Inversely correlated with lymph node metastasis	Han et al. [248], Ye et al. [249], Xiao et al. [250]
miR-139	PDE2A	Suppressor	Lung tumor	H3K27me3-mediated down-regulation of miR-139. Enhanced invasive and metastasis ability of NSCLC cells.	Watanabe et al. [251]
miR-141		Oncogene	Lung tumor	Positively associated with tumor size and, lymph node metastasis.	Zhang et al. [252]
miR-145	AEG-1/MTDH RIOK2, NOB1 N-cadherin	Suppressor	Lung tumors	Inhibited cell invasion and migration	Wang et al. [253], Liu et al. [254], Gan et al. [255], Mo et al. [84], Li et al. [204]
miR-146a		Suppressor	Serum	Lower serum level in NSCLC patients.	Wu et al. [208]
miR-147		Suppressor	Lung tumor, Serum	Low serum miR-147 expression level was correlated with lymph node metastasis and worse OS.	Chu et al. [256]
miR-148a	ROCK1 Wnt1	Suppressor	Lung tumors	Reduced cell invasion and inhibited EMT	Li et al. [89], Chen et al. [257], Chen et al. [258], Li et al. [259]
miR-148b		Suppressor	Lung tumors	High miR-148b expression had a favorable prognosis.	Ge et al. [260]
miR-150		Oncogene	Lung tumors	High miR-150 expression had a poor prognosis.	Yin et al. [261]
miR-153	ADAM19	Suppressor	Lung tumors	Inhibited cell proliferation, migration, and invasion.	Shan et al. [262], Chen et al. [263]
miR-181a-5p	HMGB2	Suppressor	Lung tumors	LncRNA NEAT1 promoted proliferation and invasion by targeting miR-181a-5p.	Li et al. [264]
miR-181b		Suppressor	Lung tumors	LncRNA NEAT1 up-regulated the miR-181a-5p-targeted gene HMGB2 through acting as a competitive "sponge" of miR-181a-5p.	Yang et al. [265]
miR-183-3p		Oncogene	Lung adenocarcinoma tissues	Involved in lung cancer pathogenesis and progression, and could be used as a potential prognostic biomarker of female lung adenocarcinoma.	Xu et al. [266]
miR-185	KLF7	Suppressor	Lung tumors	Inhibited the cell propagation, cell colony formation, and incursion capacities in vitro.	Zhao et al. [267]
miR-186	Cdc42	Suppressor	Lung tumors	Inhibited cell invasion and metastasis	Dong et al. [268], Li et al. [269]
miR-193a-3p	ERBB4, S6K2 AEG-1	Suppressor	Lung tumors	Inhibited NSCLC cell migration, invasion, and EMT in vitro and lung metastasis formation in vivo.	Yu et al. [270], Ren et al. [271]
miR-193a-5p	PIK3R3 mTOR	Suppressor	Lung tumors	Inhibited NSCLC cell migration, invasion, and EMT in vitro and lung metastasis formation in vivo.	Yu et al. [272]
miR-195		Suppressor	Plasma	Decreased plasma miRNA-195 expression was associated with lymph node metastasis and advanced clinical stage	Su et al. [273]
miR-198	SHMT1	Suppressor	Lung adenocarcinoma	Inhibited cell proliferation, enhanced cell apoptosis, and led to cell-cycle arrest	Wu et al. [274]
miR-200c	USP25	Suppressor	Lung tumors	Inhibited cell invasion and migration. Negatively correlated with lymph node metastasis.	Li et al. [115], Ceppi et al. [275]
miR-200c		Oncogene	Lung tumor	Higher expression of miR-200c was associated with poor prognosis.	Si et al. [276], Liu et al. [209]
miR-202	STAT3	Suppressor	Lung tumors	Inhibited cell proliferation, migration, and invasion.	Zhao et al. [277]
miR-203	LASP-1	Suppressor	Lung tumors	LASP-1, regulated by miR-203, promoted tumor proliferation and aggressiveness in human NSCLC.	Zheng et al. [278]
miR-210		Oncogene	Lung tumor Serum	MiR-210 expression levels might be a novel diagnostic and prognostic marker of NSCLC	Osugi et al. [279], Li et al. [280]
miR-211-3p		Suppressor	Lung tumors	LncRNA SNHG15 promoted cell proliferation and migration by targeting miR-211-3p	Cui et al. [281]
miR-212	SOX4	Suppressor	Lung tumors	Suppressed cell migration and invasion, and EMT in NSCLC cells	Tang et al. [282]
miR-215	ZEB2	Suppressor	Lung tumors	Inhibited cell migration and invasion.	Hou et al. [83]
miR-218	Slug/ZEB2	Suppressor	Lung tumors	Inhibited cell migration and invasion.	Shi et al. [59]
miR-221		Oncogene	Lung tumors	Correlated with lymph node metastasis and disease progression.	Zhang et al. [283]
miR-224		Suppressor	Lung tumors	Inhibited cell proliferation, invasion, and migration, and promoted cell apoptosis.	Zhu et al. [284]
miR-301a		Oncogene	Lung tumors	miR-301a overexpression was correlated with lymph node metastasis and poor prognosis.	Shi et al. [285]
miR-302b-3p	GCNT3	Suppressor	Lung tumors	Inhibited proliferation, migration, and invasion	Li et al. [72]
miR-328-3p	γ-H2AX	Suppressor	Lung tumors	Up-regulated miR-328-3p demonstrated a survival inhibition effect in A549 and restored NSCLC cell sensitivity to radiotherapy.	Ma et al. [286]
miR-335	Bcl-w, SP1	Suppressor	Lung tumors	Inhibited cell proliferation, migration, Increased apoptosis.	Wang et al. [287]
miR-338-3p	IRS2	Suppressor	Lung tumors	Inhibited growth and invasion.	Zhang et al. [288]
miR-339-5p	BCL6	Suppressor	Lung tumors, Peripheral blood	Inhibited cell migration and invasion	Li et al. [289], Li et al. [290]
miR-340	CDK4	Suppressor	Lung tumors	Suppressed cell proliferation.	Qin et al. [291]
miR-361-3p	SH2B1	Suppressor	Lung tumors	Inhibited cell growth, proliferation, colony formation, invasion, and migration	Chen et al. [76]
miR-361-5p		Suppressor	Lung tumors	Lower miR-361-5p expression was found in NSCLC and associated lymph node metastasis.	Zhuang et al. [292]
miR-365	TTF-1	Suppressor	Serum	High miR-365 serum level had less lymph node metastasis and longer OS.	Liu et al. [293], Sun et al. [294]
miR-378	HMOX1	Oncogene	Lung tumors	Modulated NSCLC progression and angiogenesis	Skrzypek et al. [295]
Mir-379	IGF-1R	Suppressor	Lung tumors	Inhibited cell proliferation, migration, and invasion.	Zhou et al. [296]
miR-381	LRH-1	Suppressor	Lung tumors	Inhibited cell migration and invasion in vitro and in vivo.	Tian et al. [297]
miR-383		Suppressor	Lung tumors	Reduced proliferation, invasion, and migration.	Shang et al. [298]
miR-409-3p	c-MET	Suppressor	Lung adenocarcinoma tumors	Inhibited cell proliferation, induced apoptosis, and reduced invasion and migration by silencing of AKT signaling.	Wan et al. [299]
miR-411		Oncogene	Serum	Elevated serum miR-411 expression was correlated with lymph node metastasis and poor prognosis.	Wang et al. [300]
miR-421		Oncogene	Lung tumors, Serum	Promoted cell proliferation, invasion, and migration.	Li et al. [301]
miR-422a	61 potential target genes ^§^	Oncogene	Lymph nodes and plasma	High expression in NSCLC metastatic lymph nodes and validated by fresh blood of patients.	Wu et al. [302]
miR-433	E2F3	Suppressor	Lung tumors	Reduced cell proliferation and invasion	Liu et al. [303]
miR-448	DCLK1	Suppressor	Lung squamous cell carcinoma	Inhibited cell proliferation, colony formation, migration, and invasion	Shan et al. [304]
miR-449a	c-MET	Suppressor	Lung tumors	Inhibited cell migration and invasion	Luo et al. [305]
miR-451	RAB14	Suppressor	Lung tumors,	Inhibited cell proliferation and triggered apoptosis	Wang et al. [306], Wang et al. [307]
miR-451a		Oncogene	Plasma	Exosomal miR-451a showed a significant association with lymph node metastasis, vascular invasion, and stage	Kanaoka et al. [308]
miR-452	BMI1	Suppressor	Lung tumors	Inhibited cell invasion, but not cell proliferation or apoptosis.	He et al. [309]
miR-452-5p	10 hub genes^@^	Suppressor	Lung tumors, TCGA	Low miR-452-5p expression level played an essential role in lung adenocarcinoma.	Gan et al. [310]
miR-454	PTEN	Oncogene	Lung tumors	Promoted cell proliferation, invasion, and migration and inhibited apoptosis.	Zhu et al. [311]
miR-485-5p	IGF2BP2	Suppressor	Lung tumors	Inhibited cell growth, invasion, and caused G0/G1 arrest	Huang et al. [312]
miR-486-5p	ARHGAP5, Pim-1	Suppressor	Lung tumors, Sputum, plasma	Inhibited tumor progression and metastasis	Shen et al. [313], Wang et al. [314], Pang et al. [315]
miR-490-3p	PCBP1	Oncogene	Lung tumors	Promoted cell proliferation, invasion, and migration.	Li et al. [177]
miR-491-5p	IGF2BP1	Suppressor	Lung tumors	Reduced cell proliferation, colony formation, migration, and invasion	Gong et al. [316]
miR-494		Oncogene	Lung tumor, Serum	High miR-494 level was correlated with lymph node metastasis and poor prognosis.	Zhang et al. [317], Wang et al. [318]
miR-504	LOXL2	Suppressor	Lung tumors	Inhibited cell proliferation, cell invasion, and EMT process of NSCLC	Ye et al. [319]
miR-506-3p	COTL1	Suppressor	Lung tumors	Reduced cell growth, migration, and invasion in vitro and in vivo.	Guo et al. [320]
miR-520b	Rad22A	Suppressor	Lung tumors	Inhibited cell proliferation, invasion, and metastasis abilities	Zhang et al. [321]
miR-548I	AKT1	Suppressor	Lung tumors	Inhibited NSCLC cell migration and invasion.	Liu et al. [322]
miR-582-5p	MAP3K2	Suppressor	Lung tumors	Suppressed the proliferation, migration, and invasion of NSCLC cells	Wang et al. [323]
miR-588	GRN	Suppressor	Lung squamous cell carcinoma	Suppressed tumor cell migration and invasion.	Qian et al. [324]
miR-590	OLFM4	Oncogene	Lung adenocarcinoma	Promoted cell migration and invasion.	Liu et al. [94]
miR-592	SOX9	Suppressor	Lung tumors	Reduced cell proliferation, colony formation, migration, and invasion.	Li et al. [325]
miR-598	ZEB2	Suppressor	Lung tumors	Reduced NSCLC cell proliferation and invasion.	Tong et al. [92]
miR-638		Suppressor	Serum	Serum miR-638 expression levels in NSCLC patients after chemotherapy were associated with disease prognosis.	Wang et al. [326]
miR-650	ING4	Oncogene	Lung tumors	Inhibited caspase-3-dependent apoptosis	Huang et al. [327]
miR652-3p	Lgl1	Oncogene	Lung tumors	Promoted cell proliferation, invasion, and migration.	Yang et al. [328]
miR-661	RUNX3	Oncogene	Lung tumors	Down-regulation of miR-661 suppressed NSCLC proliferation and invasion.	Wang et al. [329]
miR-675-5p	GPR55	Suppressor	Lung tumors	Inhibited cell proliferation, colony formation, invasion, and migration, and attenuated the tumorigenicity in vivo.	He et al. [330]
miR-874	MMP2, XIAP	Suppressor	Lung tumors	LncRNA XLOC_008466 functioned as an oncogene in NSCLC by regulating the miR-874-MMP2/XIAP axis	Yang et al. [331]
miR-935	E2F7	Suppressor	Lung tumors	Suppressed cell proliferation, migration, and invasion	Wang et al. [332]
miR-1204	PITX1	Oncogene	Lung tumors	Promoted cell proliferation and reduced cell cycle arrest.	Jiang et al. [333]
miR-1269	TP53, Caspase-9	Oncogene	Lung tumors, TCGA	Promoted cell survival and proliferation.	Bao et al. [334]
miR-1290	IRF2	Oncogene	Lung tumors Serum	Promoted cell growth	Jin et al. [335], Mo. Et al. [336]
miR-4317	FGF9 CCND2	Suppressor	Lung tumors	Inhibited proliferation, colony formation, migration, and invasion, and hampered cycles	He et al. [178]
miR-6754-5p		Suppressor	Lung tumors	LncRNA LINC00978 promoted cell proliferation and invasion in NSCLC by inhibiting miR-6754-5p.	Li et al. [337]

* There were 24 putative target genes for Let-7g after analysis by miRanda, TargetScan, Pictar and miRDB prediction algorithms. ^#^ TargetScan software were applied for in silico prediction of miR-31 targets. ^¶^ There were 44 co-regulated target genes of both miRNA-126-3p and miRNA-126-5p by using twelve target gene prediction software programs (TargetScan, miRWalk, Microt4, miRDB, miRanda, miRBridge, miRMap, miRNAMap, PITA, PicTar2, RNA22 and RNAhybrid). ^§^ Identified by predicting by online database, miRecords and mining of the data from Gene Expression Omnibus (GEO) and TCGA. ^@^ Total of the fourteen prediction programs were used for screened the putative target genes. STRING database was used for the selection of hub genes which were probably involved in the strategic pathway related to lung adenocarcinoma. Bcl-2: B-cell lymphoma 2; PTEN: phosphatase and tensin homolog; PBMC: peripheral blood mononuclear cell; BCL11A: B-cell lymphoma/leukemia 11A; Cthrc1: collagen triple helix repeat containing 1; PLK-1: polo-like kinase 1; Mcl-1: myeloid cell leukemia 1; lncRNA SNHG1: long non-coding RNA small nucleolar RNA host gene 1; BDNF: brain-derived neurotrophic factor; NEDD9: neural precursor cell expressed, developmentally down-regulated 9; PDK1: 3-phosphoinositide-dependent protein kinase-1; Sirt1: silent mating type information regulation 2 homolog 1; YAP1: Yes associated protein 1; AEG-1: astrocyte elevated gene-1; MTDH: metadherin; RIOK2: right open reading frame kinase 2; NOB1: nin one binding protein; KLF7: Kruppel-like factor 7; PTTG1: pituitary tumor-transforming 1; Cdc42: cell division control protein 42 homolog; ERBB4: erb-b2 receptor tyrosine kinase 4; S6K2: S6 kinase 2; PIK3R3: phosphatidylinositol 3-kinase, regulatory subunit 3; mTOR: mammalian target of rapamycin; SHMT1: serine hydroxymethyltransferase 1; USP25: ubiquitin-specific protease 25; LASP-1: LIM and SH3 protein 1; GCNT3: glucosaminyl (N-acetyl) transferase 3, mucin type; γ-H2AX: phosphorylated histone H2AX; Bcl-w: B-cell lymphoma 2 like 2; SP1: specificity protein 1; IRS2: insulin receptor substrate 2; Bcl-6: B-cell lymphoma 6; CDK4: cyclin-dependent kinase 4; SH2B1: Src homology 2B (SH2B) family members 1; HMOX1: heme oxygenase-1; IGF-1R: insulin-like growth factor 1 receptor; LRH-1: liver receptor homolog-1; E2F3: human E2F transcription factor 3; DCLK1: doublecortin-like kinase 1; c-MET: hepatocyte growth factor receptor; RAB14: ras-related protein 14; BMI1: B lymphoma Mo-MLV insertion region 1; IGF2BP2: insulin-like growth factor 2 mRNA-binding protein 2; ARHGAP5: Rho GTPase-activating protein 5; Pim-1: proviral integration site 1; PCBP1: poly(RC) binding protein 1; IGF2BP1: insulin-like growth factor 2 mRNA-binding protein 1; LOXL2: lysyl oxidase-like 2; COTL1: coactosin-like protein; Rad22A: DNA repair and recombination protein rad22; PDE2A: Phosphodiesterase 2A; MAP3K2: mitogen-activated protein kinase kinase kinase 2; GRN: progranulin; OLFM4: olfactomedin 4; ING4: inhibitor of growth 4; Lgl1: late gestation lung protein 1; RUNX3: runt-related transcription factor 3; GPR55: G protein-coupled receptor 55; XIAP: X-linked inhibitor of apoptosis protein; E2F7: Homo sapiens E2F transcription factor 7; PITX1: paired like homeodomain 1; TP53: tumor protein p53; IRF2: interferon regulatory factor 2; FGF9: fibroblast growth factor 9; CCND2: cyclin D2.

**Table 5:** MicroRNA-targeted therapy for lung cancer.

Phase of Drug Development	Year	miRNA	Target	Delivery System	Author/Reference
In vitro	2010	miR-145	C-MYC	Lentivirus	Chen et al. [359]
In vitro, In vivo	2012	miR-145	OCT4, SOX2	Polyethylenimines	Chiou et al. [360]
In vitro, In vivo	2010	miR-126	EGFL7	Lipid	Sun et al. [358]
In vitro, In vivo	2010	Let-7	KRAS	Lentivirus	Trang et al. [355]
In vitro, In vivo	2010, 2011	miR-34a	BCL-2	Neutral lipid	Wiggins et al. [356], Trang et al. [272]
In vitro, In vivo	2011, 2015	Let-7, miR-34a	KRAS, TP53	Lentivirus, Lipid	Trang et al. [272], Stahlhut et al. [357]
In vitro, In vivo	2011	miR-133b	MCL-1	Cationic lipoplex	Wu et al. [361]
In vitro, In vivo	2013	miR-29b	MCL-1, CDK6, DNMT3	Lipid	Wu et al. [362]
Phase I (NCT01829971)	Start: 2013 Termination: 2016	miR-34a	BCL-2	Lipid	
Phase I (NCT02369198)	2017	miR-16	EGFR	Bacterial minicells	van Zandwijk et al. [366]

Oct4: octamer-binding transcription factor 4, Sox2: sex determining region Y-box 2; EGFL7: epidermal growth factor–like domain 7; KRAS: Kirsten rat sarcoma 2 viral oncogene homolog; Bcl-2: B-cell lymphoma 2; TP53: tumor protein P53, Mcl-1: myeloid cell leukemia 1; CDK6: cell division protein kinase 6; DNMT3: DNA methyltransferase 3; EGFR: epidermal growth factor receptor.
